# Employee perceived overqualification and innovation performance: the roles of self-oriented perfectionism and job crafting

**DOI:** 10.3389/fpsyg.2024.1398163

**Published:** 2024-08-07

**Authors:** Bing Jiang, Hongxin Qiu, Siyi Liu, Ji Zhang

**Affiliations:** ^1^School of Management, Shandong University of Technology, Zibo, China; ^2^School of Economics, Shandong University of Technology, Zibo, China

**Keywords:** perceived overqualification, self-oriented perfectionism, job crafting, innovation performance, independent self-construction, informal status

## Abstract

Leveraging the trait activation theory, the study constructs a model featuring moderated chain mediation to explore how perceived overqualification influences employee innovation performance. After conducting two surveys with Chinese employees, this study collects 363 valid questionnaires. The findings reveal that perceived overqualification is positively related to employee innovation performance. Both self-oriented perfectionism and job crafting are partial mediators between perceived overqualification and innovation performance, and they collectively play a chain mediating role. Furthermore, independent self-construction positively moderates the link between perceived overqualification and self-oriented perfectionism, and informal status positively moderates the relationship between job crafting and employee innovation performance. Additionally, the indirect influence of perceived overqualification on employee innovation performance is moderated by independent self-construction and informal status. This study adds to the current body of literature on perceived overqualification and offers practical implications for organizations aiming to enhance innovation performance.

## 1 Introduction

In recent years, various factors, such as the global economic recession, advancements in education, and a challenging employment landscape, have contributed to a scenario where numerous professionals' educational backgrounds, skills, and social experience surpass the demands of available positions. This phenomenon, widely observed in the global labor market, is collectively known as overqualification (Zhang et al., 2016). According to a report, nearly half of the worldwide workforce (47%) regards their qualifications as exceeding the requirements for their current positions, with particularly pronounced sentiments in China (84%) (Lin et al., 2017). Perceived overqualification is often associated with reduced job satisfaction and negative workplace behaviors (Cheng et al., 2020). However, it is noteworthy that, through specific mechanisms or within particular contexts, individuals with perceived overqualification can distinguish themselves within an organization and exhibit positive work behaviors (Zhang et al., 2021). Such behaviors prove advantageous for the organization's long-term development. Consequently, exploring how to leverage perceived overqualification to enhance employees' work output becomes imperative.

Prior research has generally ascertained that perceived overqualification yields more adverse employee effects. For instance, individuals harboring a perception of being overqualified tend to experience diminished job satisfaction, affective commitment, and other work attitudes (Lobene and Meade, 2013; Harari et al., 2017), leading to negative work behaviors such as job withdrawal, cyberloafing, and voluntary separation (Cheng et al., 2020; Zhang et al., 2020; Ma and Zhang, 2022). Recent research has begun shedding light on the positive aspects associated with perceived overqualification. For instance, Hu et al. (2015) found that employees who perceive themselves as overqualified tend to attribute higher importance to their tasks, which subsequently leads to increased levels of organizational citizenship behaviors. Deng et al. (2018) proposed that perceived overqualification can enhance employees' career commitment. Ma et al. (2020) posited that employees with elevated perceived overqualification often manifest higher levels of positive self-perceived resources, consequently displaying more favorable work behaviors. Despite these insights, a scarcity of empirical evidence regarding the positive effects of perceived overqualification persists (Dar and Rahman, 2020), impeding a comprehensive understanding of its overall impact. Innovation is a primary avenue for employees to cultivate core competitive advantages and actualize enhanced life value, representing a crucial manifestation of their work initiative. Despite existing attention to the influence of perceived overqualification on employee innovation (e.g., perceived overqualification is positively related to employee creativity; Liu and Wang, 2012), a study has yet to elucidate this relationship from a trait activation perspective. Innovation performance, delineating the cumulative outcomes of the employee innovation process, serves as a metric for gauging the results and effectiveness of their innovative activities (Mumford, 2000). Given the prevalence of the “underutilization of abilities” phenomenon in China, this study investigates the impact of perceived overqualification on employee innovation performance within the framework of trait activation theory in the context of Chinese management. The primary focus is to unveil the underlying mechanisms and boundary conditions associated with this indirect influence.

Perceived overqualification engenders a pervasive sense among employees that their skills and qualifications are underappreciated and underutilized, making them prone to negative emotions (Zhang et al., 2020). In psychology, it is a fundamental principle that individuals seek to maximize positive experiences and minimize negative ones (Alicke and Sedikides, 2009). When employees perceive themselves as overqualified, they may develop an intrinsic drive to prove their worth through superior performance. This intrinsic motivation can propel them to excel in their work, thereby enhancing their innovation performance. Consequently, self-oriented perfectionism, characterized by a strong motivation to pursue high standards and goals (Kim et al., 2022), is likely to serve as a bridge between perceived overqualification and employee innovation performance. Motivated by self-oriented perfectionism, employees channel their resources toward innovative endeavors and embrace job crafting behaviors involving transforming, designing, and enhancing job aspects to attain higher professional objectives. Throughout the job crafting process, employees continually improve their innovation abilities through learning and reflective summarization (Tims et al., 2014), resulting in an ultimately favorable impact on innovation performance. Furthermore, the influence of perceived overqualification on employees' self-oriented perfectionism exhibits variation based on individual characteristics. Those strongly inclined toward independent self-construction prioritize personal development and skill enhancement (Lacko et al., 2021), providing a foundation for generating self-oriented perfectionism. Additionally, the recognition and influence derived from informal status significantly contribute to employees' reassessment of their self-work and job crafting, thereby enhancing innovation performance (Wang and Lau, 2022). Consequently, independent self-construction and informal status play pivotal moderating roles in shaping the trajectory of employee innovation performance improvement.

In summary, the study endeavors to investigate the mechanisms and boundary conditions that underlie the impact of perceived overqualification on employee innovation performance. Firstly, the study scrutinizes the impact of perceived overqualification on employee innovation performance within the Chinese management context by applying the conservation of resource theory. Secondly, the research delves into the pathways of perceived overqualification in enhancing innovation performance, examining the dual perspectives of self-oriented perfectionism and job crafting. Thirdly, the study analyzes the moderating effects of independent self-construction and informal status in the relationship between perceived overqualification and innovation performance. Lastly, a moderated chain mediation model is proposed, systematically elucidating the influence mechanism and boundary conditions of perceived overqualification on employee innovation performance. This endeavor aims to broaden our understanding of the positive outcomes and mechanisms associated with perceived overqualification. Additionally, it aims to offer insights and recommendations for enterprises in guiding employees with a sense of overqualification to unleash their creative potential.

## 2 Theory and hypotheses

### 2.1 Employee perceived overqualification and innovation performance

Perceived overqualification pertains to an individual's belief that their education, experience, and skills surpass the requisites of their current job (Maynard et al., 2006), which essentially constitutes a subjective evaluation and feeling that the individual's abilities and quality are not optimally aligned with the job. The perception of perceived overqualification comprises two central elements. First is the condition of surplus qualification, evident as a directional “demand-ability” mismatch (Debus et al., 2020). Second, it involves employees' subjective awareness of the disparity between their qualifications and the job requirements. Employees frequently gauge their performance against their ideal self-image and the accomplishments of peers in analogous roles. This subjective comparison engenders personal cognition and elicits emotional responses from employees. Thus, perceived overqualification is rooted in objective overqualification rather than subjective overconfidence stemming from unwarranted beliefs.

Innovation performance denotes the systematic generation of innovative ideas and the implementation of inventive actions within the organizational context (Wang and Chen, 2022). This process facilitates employees in augmenting their intrinsic value, enabling them to navigate the dynamic organizational environment effectively. Successful innovation performance necessitates not only profound knowledge and exceptional skills but also the subjective initiative of employees. Initially, perceived overqualification propels employees to perceive routine tasks as inadequate for fulfilling their value pursuit. Consequently, they allocate time and effort toward seeking challenges and innovations to satisfy internal aspirations and augment their self-esteem, ultimately fostering innovation performance (Dar and Rahman, 2020). Moreover, employees with perceived overqualification consider their surplus knowledge and abilities valuable and scarce resources within the organization. Drawing from the conservation of resource theory (Hobfoll et al., 2018), employees who want to preserve and cultivate these advantageous resources will exert additional efforts to acquire new skills, initiate novel projects, and employ diverse strategies and methods in their work. This heightened engagement increases the likelihood of organizational innovation performance. Lastly, when employees perceive themselves as more competent than their peers, they exhibit heightened confidence. This confidence mitigates their fear of innovation failure, empowering them to explore novel approaches and thereby enhance innovation performance. Building upon these observations, we posit the subsequent hypotheses:

Hypothesis 1 Perceived overqualification is positively related to employee innovation performance.

### 2.2 The mediating role of self-oriented perfectionism

Self-oriented perfectionism entails an individual's pursuit of high standards and goals, driven by intrinsic motivation to achieve perfection (Hewitt and Flett, 1991). It is associated with positive outcomes such as dedication and innovative behavior (Childs and Stoeber, 2010; Chang et al., 2016), emphasizing excellence and a zero-tolerance attitude toward imperfections (Otto et al., 2021). According to trait activation theory, individuals' traits are accentuated in specific motivational contexts, leading them to focus on situational factors aligned with their traits (Tett and Guterman, 2000). Compared to employees whose qualifications match their job requirements, those who perceive themselves as overqualified possess a unique advantage. When they recognize that their abilities exceed the demands of their current role, they proactively seek job opportunities that better align with their skills. Driven by a motivation to realize their full potential, they strive to perform their work perfectly, viewing it as an opportunity to showcase their capabilities. Therefore, this study suggests that employees with a strong perceived overqualification are likely to leverage their strengths fully, pursuing higher personal and work goals, thereby fostering self-oriented perfectionism.

Self-oriented perfectionism emphasizes employees' persistent pursuit of perfection, enabling them to actively cope with the pressure arising from job-role mismatches and demonstrate their problem-solving abilities. This, in turn, stimulates positive performance behaviors and enhances innovation performance. First, employees with self-oriented perfectionism exhibit higher work motivation and a stronger sense of efficacy (Kim et al., 2017). These personal resources facilitate the active exploration of new methods and solutions to overcome work obstacles, thereby increasing the likelihood of organizational innovation. Second, employees with high self-oriented perfectionism pursue excellence and perfection when setting and implementing work goals. During the execution of innovation strategies, they maintain this pursuit of perfection, setting higher standards and more stringent self-requirements, and integrating innovative ideas and technologies into their activities, thereby improving innovation performance. Building upon these analyses, we posit the following hypotheses:

Hypothesis 2 Self-oriented perfectionism acts as a mediator linking perceived overqualification to employee innovation performance.

### 2.3 The mediating role of job crafting

Job crafting pertains to employees spontaneously reshaping their roles to better align with their abilities and interests (Dutton, 2001). Employees perceiving themselves as overqualified often demonstrate the capability to surpass job requirements, leading to a positive self-evaluation. However, the acknowledgment of employees' “positive selves” by leaders and colleagues is typically confined to the assessment of job duties. Consequently, the self-image of overqualified employees goes unrecognized in their current roles, motivating them to engage in job redesign efforts to uphold a positive self- perception (Lin et al., 2017). This motivation is highly likely to foster job-crafting behaviors. Therefore, job crafting, characterized as an active behavior optimizing the alignment between employees and their roles, should be considered an outcome of perceived overqualification.

Job crafting is an innovative learning process wherein employees reevaluate the significance of their jobs and redesign their content (Zhang and Parker, 2019), reflecting a series of behavioral manifestations to achieve superior job performance. Firstly, employees engaged in extensive job crafting exhibit heightened positivity toward their work, demonstrating proactive and adaptive behaviors (Petrou et al., 2012; Guo and Hou, 2022). This proactivity encourages them to seek opportunities for job breakthroughs or actively accept challenging tasks to implement job crafting (Kapica and Baka, 2021) and stimulate more innovative behavior. Secondly, job crafting grants employees greater autonomy and decision-making authority, stimulating their innovation awareness and motivating them to experiment with novel approaches and ideas, thereby enhancing innovation performance. Lastly, through job crafting, employees garner increased recognition from others, enhancing cooperative relationships and creating favorable conditions for implementing innovative behaviors (Erdogan et al., 2020). Furthermore, employees bolster their self-efficacy through learning and reflective thinking to summarize their work experience in the process of job crafting, enhancing their ability to implement innovative behaviors and ultimately improving their innovation performance. Building upon these observations, we formulate the following hypotheses:

Hypothesis 3 Job crafting acts as a mediator linking perceived overqualification to employee innovation performance.

### 2.4 The chain mediation of self-oriented perfectionism and job crafting

Self-oriented perfectionism reflects employees' cognitive pursuit of self-perfection and self-improvement. To achieve perfection and avoid uncertain or flawed outcomes, employees high in self-oriented perfectionism exert significant effort to overcome difficulties and meet high standards (Chang et al., 2016). This relentless pursuit of perfection drives them to continuously examine and reflect on their work methods, discard inefficient procedures, and proactively engage in transformative job crafting aligned with their self-actualization goals. This process not only enhances organizational recognition and personal development but also increases job satisfaction. Moreover, employees with self-oriented perfectionism exhibit strong initiative and a notable emphasis on prioritization and ego in their work (Hewitt and Flett, 1991). When job performance does not meet their expectations, they take the initiative to craft their job to improve effectiveness. Consequently, this study concludes that self-oriented perfectionism can promote job crafting.

Trait activation theory posits that individual behavior results from a combination of personality traits and situational factors (Tett and Guterman, 2000). The perception of overqualification arises when employees believe their qualifications exceed the requirements of their current position, reflecting the traits of those who perceive overqualification. Employees with a high perception of overqualification feel their knowledge and abilities are underutilized, leading them to have higher expectations of their intellectual resources. This accelerates the formation of self-oriented perfectionism, driving them to set higher work goals. Through job crafting, they improve work efficiency and stimulate innovative behaviors, thereby enhancing their own innovation performance. Building on these insights, we advance the following hypotheses:

Hypothesis 4 Self-oriented perfectionism and job crafting act as chain mediators linking perceived overqualification to employee innovation performance.

### 2.5 The moderating influence of independent self-construction

Self-construction encompasses an individual's perception of interpersonal connections (Abele et al., 2021) and is delineated into independent and dependent self-construction (Ilies et al., 2011). Those with dependent self-construction seek positive interpersonal relationships and a sense of belonging, while individuals with independent self-construction aspire to distinctiveness and personal accomplishment. In the contemporary era, the emphasis on independent self-construction among employees is growing, profoundly influencing their work attitudes and behaviors. On one hand, independent self-constructed employees typically exhibit high self-assurance, believing in their ability to create greater value at work. When faced with perceived overqualification, they are more inclined to leverage their excess qualifications to achieve higher work goals, viewing this as an opportunity to validate their self-worth, thereby stimulating self-oriented perfectionism. On the other hand, those with high independent self-construction prioritize success and gains, prioritizing factors such as increased autonomy and adequate qualifications. These anticipated gains and positive elements also provide psychological support for their self-oriented perfectionism. Conversely, employees with low independent self-construction are inclined toward a “compromise” approach in conflict resolution. Even if they recognize that their qualifications surpass job demands, they suppress the pursuit of loftier goals to maintain a harmonious relationship with supervisors, diminishing the likelihood of generating self-oriented perfectionism. Based on these insights, we posit the following hypotheses:

Hypothesis 5 The positive association between perceived overqualification and self-oriented perfectionism is moderated by independent self-construction.

Moreover, the impact of perceived overqualification on employee innovation performance, mediated by self-oriented perfectionism and job crafting, is contingent on the degree of independent self-construction. Employees with a heightened sense of independent self-construction tend to exhibit more incredible foresight, self-esteem, and creativity (Oeberst and Wu, 2015), making them more prone to initiating self-oriented perfectionism in response to perceived overqualification. This, in turn, motivates employees to reconfigure their job responsibilities, aiming for heightened innovation performance. In contrast, employees with low levels of independent self-construction tend to depend on others for guidance and are hesitant to assume additional responsibilities and challenges. Consequently, they struggle to develop self-oriented perfectionism when faced with perceived overqualification. Constrained by low levels of independent self-construction, these employees lack the intrinsic drive to pursue career advancement and are less likely to engage in job crafting behaviors, consequently diminishing their innovation performance. Building on these insights, we posit the following hypotheses:

Hypothesis 6 The chain-mediated connection between perceived overqualification, self-oriented perfectionism, job crafting, and employee innovation performance is moderated by independent self-construction.

### 2.6 The moderating influence of informal status

Informal status pertains to an individual's recognition from other members within an organization based on informal channels (Zhang et al., 2023). It encompasses the esteem, prestige, and influence that an individual commands in the perception of their peers. Employees with higher informal status typically occupy more favorable embedded positions within the organization, assuming leadership roles in relationships and interactions with other members. This positioning facilitates resource mobilization and external support, critical for the smooth progression of job crafting processes. Consequently, individuals with elevated informal status levels possess distinct advantages in achieving high innovation performance outputs through job crafting behaviors. Drawing from social identity theory (Spears, 2021), individuals are inclined to uphold their reputation by displaying traits aligning with high status, deriving psychological incentives from the respect and admiration garnered from others, thereby fostering status-protective motives. For employees with high informal status, maintaining their esteemed position motivates them to challenge existing work modes and processes through remodeling, fostering self-reflection and creativity stimulation (Wang and Lau, 2022), thereby enhancing innovative performance. Conversely, employees with lower informal status face limited resources and control. Even with innovative ideas for work improvement, implementing them becomes challenging without long-term recognition and support for their proposals. This scenario impedes the enhancement of their ultimate innovation performance. Grounded in these observations, we posit the following hypothesis:

Hypothesis 7 The positive association between job crafting and employee innovation performance is moderated by informal status.

Drawing further implications from the preceding analysis, informal status moderates the chain-mediated relationship where perceived overqualification propels employee innovation performance. Higher informal status endows employees with additional intangible resources, including trust and work autonomy, fostering increased organizational cooperation and support. Consequently, this heightened status holds more significant sway over organizational development and operations (Magee and Galinsky, 2008). Specifically, employees experiencing pronounced perceived overqualification, bolstered by informal status, are more prone to engaging in challenging appraisals of stressful situations. They leverage their extensive experiential knowledge to recalibrate their current work content, fostering an environment conducive to enhancing innovation performance. Conversely, employees with lower informal status often face a lack of recognition for their work and contributions, leading to reduced motivation, engagement, and potential feelings of depression. In such circumstances, even if employees perceive themselves as overqualified, they struggle to engage in challenging appraisals due to a negative mindset. This reluctance significantly hampers their ability to engage in job crafting behaviors, thereby failing to provide the necessary support for enhancing innovation performance. Grounded in these insights, we posit the following hypotheses:

Hypothesis 8 The chain-mediated connection between perceived overqualification, self-oriented perfectionism, job crafting, and employee innovation performance is moderated by informal status.

**Figure 1:**
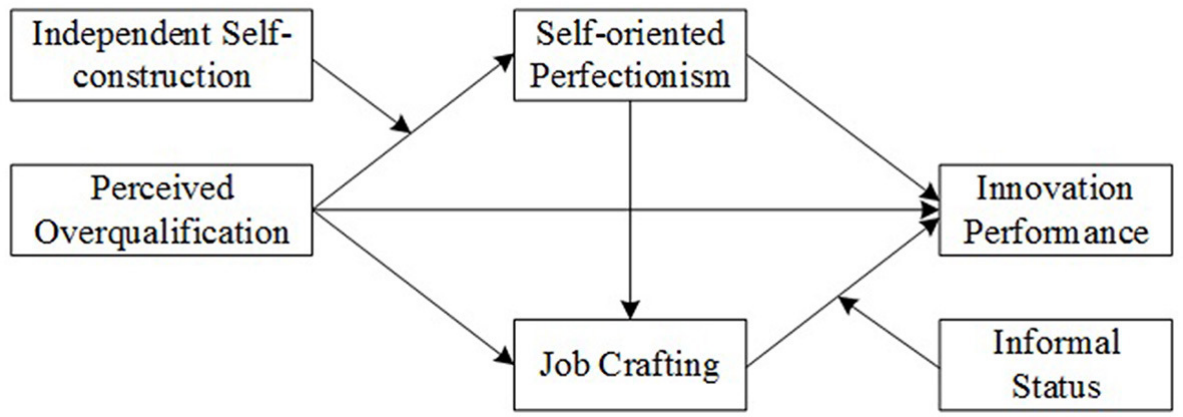
Theoretical model.

In conclusion, Figure 1 illustrates the theoretical model of the study.

## 3 Methods

### 3.1 Data collection and sample analysis

The study participants were from 6 operational enterprises in China, encompassing both manufacturing and service sectors. Before conducting the survey, we established contact with these enterprises' Human Resource (HR) managers through our network, outlining the research's objectives. With the endorsement of the human resource management departments, 80 employees were randomly selected from each enterprise, and paper questionnaires were distributed with a specified response deadline. To safeguard the authenticity of survey data, employees were instructed to furnish the final five digits of their mobile phone numbers following the completion of each questionnaire, facilitating the identification of survey subjects. Moreover, it was emphasized that the questionnaire data were solely designated for academic research purposes. Employees were made aware of their option to conclude or withdraw from the survey at any point. To mitigate homophily bias, our questionnaire was designed longitudinally. The initial phase primarily involved the collection of responses from employees on perceived overqualification, self-oriented perfectionism, job crafting, and demographic information, including gender, age, education, and tenure. The subsequent phase focused on gathering responses about innovation performance, independent self-construction, and informal status. Upon completing the questionnaire collection, we would meticulously review the contents. In total, 480 questionnaires were distributed across the two surveys, and 363 were deemed as final valid submissions, resulting in a commendable questionnaire validity rate of 72.6%.

Among the 363 surveyed employees, 59% are male. On average, participants have an age of 34.66 years (SD = 7.55), and their tenure is 30.33 months (SD = 9.15). Regarding educational attainment, bachelor's and master's degrees predominated, accounting for 38.7% and 51.8%, respectively.

### 3.2 Measures

The research model encompasses six variables: perceived overqualification, self-oriented perfectionism, job crafting, innovation performance, independent self-construction, and informal status. All scales employed in this study are derived from well-established instruments found in prior literature. Responses utilized a five-point Likert scale, responding on a range from 1 to 5, where 1 signifies “completely disagree,” and 5 denotes “completely agree.”

#### 3.2.1 Perceived overqualification

The evaluation of perceived overqualification employs the scale crafted by Maynard et al. (2006), which comprises seven items, this measure assesses perceived overqualification in terms of knowledge, education, and skills. Representative items include “My level of qualification is higher than the level of qualification required by the job.” and “Much of my knowledge is not useful in my current job.”

#### 3.2.2 Self-oriented perfectionism

The evaluation of self-oriented perfectionism is based on the perfectionism scale developed by Hewitt and Flett (1991). This scale includes eight items specifically focused on self-oriented perfectionism. Representative items are “At work, I set high standards for myself.” and “At work, I try to be perfect.”

#### 3.2.3 Job crafting

The evaluation of job crafting employs a fifteen-item scale crafted by Slemp and Vella-Brodrick (2014), encompassing three dimensions: task crafting, cognitive crafting, and relational crafting. This scale is adapted to suit the assessed group. Representative items include “I would implement novel approaches to enhance my work.” and “I proactively adjust the type or scope of tasks to do my job better.”

#### 3.2.4 Innovation performance

The evaluation of innovation performance is based on the scale crafted by Janssen and Van Yperen (2004), comprising three dimensions: idea generation, idea facilitation, and idea realization. This scale includes a total of nine items. Representative items include “I can gain organizational recognition for my innovative ideas.” and “I can implement innovative ideas effectively.”

#### 3.2.5 Independent self-construction

The evaluation of independent self-construction is based on the self-construction scale crafted by Brockner et al. (2000), with five items selected to assess independent self-construction. Representative items include “I take pleasure in expressing uniqueness in diverse aspects compared to others.” and “My personality trait is that I do not want to be constrained by others.”

#### 3.2.6 Informal status

Informal status is evaluated through a three-item scale assessing workplace status, derived from the Workplace Status Scale developed by Djurdjevic et al. (2017). Representative items include “I have a high status in the organization.” and “I have a high reputation in the organization.”

#### 3.2.7 Control variables

Drawing upon extant literature (Wadei et al., 2021), gender, age, education, and tenure are employed as control variables to mitigate the influence of these factors on self-oriented perfectionism, job crafting, and innovation performance. Gender (1 = male, and 2 = female) and education (1 = junior college or below, 2 = undergraduate degree, 3 = master's degree, and 4 = doctoral degree or above) test entries utilized Likert's five-point scoring method, while age (in years) and tenure (in months) are self-reported.

### 3.3 Analytic strategy

In this study, SPSS 26.0 was utilized for data analysis, while AMOS 24.0 was employed for confirmatory factor analysis and common method bias testing. Firstly, Cronbach's α and composite reliability (CR) were used to assess the reliability of the scale. Secondly, confirmatory factor analysis was conducted to evaluate the discriminant validity of the main variables. Thirdly, common method bias was tested using Harman's single factor analysis and the addition of a common method variance (CMV) factor. Fourthly, an analysis of Pearson correlations was undertaken to explore the connections between the primary variables. Lastly, the hypotheses underwent scrutiny through hierarchical regression analysis combined with the bootstrap method.

## 4 Results

### 4.1 Reliability and validity test

The reliability and validity test results for each variable are presented in Table 1. The Cronbach's α and composite reliability (CR) values for all six variables exceed 0.8, indicating strong reliability for each scale. Validity was assessed through convergent and discriminant validity. The results show that the factor loadings for each variable were above 0.5, the KMO values exceeded 0.7, and the average variance extracted (AVE) ranged from 0.514 to 0.731, all above the threshold of 0.5. These findings indicate that the scale demonstrates good convergent validity.

**Table 1:** Reliability and validity test results.

**Variables**	**Factor loading**	**KMO**	**Cronbach's α**	**CR**	**AVE**
Perceived overqualification	0.764–0.824	0.942	0.918	0.921	0.624
Self-oriented perfectionism	0.697–0.851	0.703	0.897	0.909	0.626
Job crafting	0.610–0.963	0.871	0.838	0.939	0.514
Innovation performance	0.730–0.826	0.907	0.897	0.927	0.584
Independent self-construction	0.711–0.902	0.881	0.846	0.931	0.731
Informal status	0.772–0.862	0.702	0.807	0.862	0.677

Confirmatory factor analysis was conducted on the variables, with results presented in Table 2. The six-factor model demonstrates the best fit (χ^2^/df = 1.526, TLI = 0.986, CFI = 0.993, RMSEA = 0.018, SRMR = 0.036), indicating strong discriminant validity for the scale used in this study. Additionally, the square root of the average variance extracted (AVE) for each variable is greater than the correlation coefficients between the variables (as shown in Table 3), further confirming the good discriminant validity of the scale.

**Table 2:** Confirmatory factor analysis results.

**Models**	***χ2/df***	**Δ*χ2/df***	**TLI**	**CFI**	**RMSEA**	**RMR**
Six-Factor Model (POQ; CA; JC; ISC; IS; IP)	1.526	–	0.986	0.993	0.018	0.036
Five-Factor Model (POQ; CA + JC; ISC; IS; IP)	2.564	0.302	0.836	0.896	0.063	0.117
Four-Factor Model (POQ; CA + JC+ISC; IS; IP)	3.612	0.737	0.787	0.790	0.070	0.121
Three-Factor Model (POQ; CA + JC+ISC+IS; IP)	5.101	0.976	0.716	0.749	0.091	0.135
Two-Factor Model (POQ+ CA+ JC+ISC+IS; IP)	5.804	1.356	0.665	0.636	0.114	0.147
One-Factor Model (POQ+ CA+ JC+ISC+IS+ IP)	8.194	1.745	0.442	0.534	0.141	0.160
Six-Factor Model + CMV	1.413	3.500	0.991	0.996	0.015	0.031

N = 363; POQ represents perceived overqualification; SP represents self-oriented perfectionism; JC represents job crafting; ISC represents independent self-construction; IS represents informal status; IP represents innovation performance; “+” denotes the amalgamation of two factors into a single factor.

**Table 3:** Descriptive statistics of variables.

**Variables**	**1**	**2**	**3**	**4**	**5**	**6**	**7**	**8**	**9**	**10**
1 Gender										
2 Age	0.041									
3 Education	0.006	0.154^**^								
4 Tenure	0.017	0.882^**^	0.192^**^							
5 POQ	0.019	−0.025	−0.018	−0.066	**0.790**					
6 SP	−0.065	−0.124^*^	−0.137^*^	−0.187^**^	0.198^**^	**0.791**				
7 JC	0.042	−0.074	−0.063	−0.097	0.521^**^	0.219^**^	**0.717**			
8 IP	0.009	−0.222^**^	−0.082	−0.329^**^	0.453^**^	0.345^**^	0.437^**^	**0.764**		
9 IS	0.104	0.088	0.022	0.121^*^	−0.222^**^	0.156^**^	−0.163^**^	−0.276^**^	**0.855**	
10 ISC	0.019	0.129^*^	0.037	0.116^*^	−0.079	0.129^**^	−0.104^*^	−0.114^*^	0.027	**0.823**
M	1.580	34.660	2.110	30.330	3.817	3.668	3.749	3.709	3.891	3.033
SD	0.500	7.550	0.927	9.150	0.892	0.872	0.841	0.727	0.772	0.785

N = 363; POQ represents perceived overqualification; SP represents self-oriented perfectionism; JC represents job crafting; ISC represents independent self-construction; IS represents informal status; IP represents innovation performance. ^*^Represents p < 0.05, ^**^represents p < 0.01.

### 4.2 Test of common method variance

To mitigate the influence of common method bias, a two-stage data collection process was employed. Nevertheless, given that the variables were answered by the same subjects, there remains a potential risk of common method bias. To control for this, a common method latent factor (CMV) was added to the six-factor model following Podsakoff et al.'s (2003) suggestion. The results, presented in Table 2, indicate that while the fitting index improved slightly after adding the CMV, the overall coefficient did not change significantly (Δχ^2^/df = 3.500, p > 0.05). Therefore, common method bias is unlikely to cause serious interference with the hypothesis testing.

### 4.3 Descriptive statistical analysis

The analysis results, encompassing mean values, standard deviations, and correlation coefficients among variables, are presented in Table 3. Perceived overqualification exhibits a significant positive correlation with self-oriented perfectionism, job crafting, and innovation performance (r = 0.198, *p* < 0.01; r = 0.521, *p* < 0.01; r = 0.453, *p* < 0.01). Additionally, self-oriented perfectionism demonstrates a significant positive association with both job crafting and innovation performance (r = 0.219, *p* < 0.01; r = 0.345, *p* < 0.01), while job crafting demonstrates a positive association with innovation performance (r = 0.437, *p* < 0.01). These findings offer initial support for the hypotheses posited in this study.

### 4.4 Hypotheses testing

In this study, the hierarchical regression method (Baron and Kenny, 1986) is used to test the main effect and the intermediate effect. The outcomes are presented in Table 4. Initially, we assess the main effect. Perceived overqualification significantly contributes to positive outcomes in employee innovation performance (Model 6, r = 0.347, *p* < 0.001) while controlling for gender, age, education, and years of experience, thereby confirming Hypothesis H1. Secondly, this study examines the mediating effects of self-oriented perfectionism and job crafting. The results in Table 4 reveal that perceived overqualification significantly positively influences self-oriented perfectionism and job crafting (Model 2, r = 0.193, *p* < 0.001; Model 4, r = 0.488, *p* < 0.001). When both perceived overqualification and self-oriented perfectionism are incorporated into the regression equation, self-oriented perfectionism significantly and positively influences employee innovation performance (Model 7, r = 0.205, *p* < 0.001), confirming Hypothesis 2. Similarly, when incorporating perceived overqualification and job crafting into the regression equation, it is observed that job crafting significantly and positively influences employee innovation performance (Model 8, r = 0.219, *p* < 0.001), supporting Hypothesis 3.

**Table 4:** Results of hierarchical regression analysis.

	**Self-oriented perfectionism**		**Job crafting**		**Innovation performance**			
	**M 1**	**M 2**	**M 3**	**M 4**	**M 5**	**M 6**	**M 7**	**M 8**
Gender	−0.115	−0.121	0.072	0.059	0.012	0.001	0.018	−0.013
Age	−0.022	−0.021	0.036	−0.029	−0.024^**^	0.178^*^	0.179^**^	0.185^**^
Education	−0.017	−0.014	−0.041	−0.040	−0.046	−0.011	−0.011	−0.002
Tenure	0.004^*^	0.003^*^	−0.099	−0.020	0.011	−0.345^***^	−0.313^***^	−0.341^***^
POQ		0.193^***^		0.488^***^		0.347^***^	0.327^***^	0.240^***^
SP							0.205^***^	
JC								0.219^***^
R^2^	0.032	0.071	0.014	0.279	0.037	0.308	0.294	0.354
Δ R^2^	0.032	0.039	0.014	0.265	0.037	0.179	0.056	0.046
F-value	2.941	5.441	1.243	27.598	3.455	31.765	24.747	32.547

N = 363; POQ represents perceived overqualification; SP represents self-oriented perfectionism; JC represents job crafting. ^*^Represents p < 0.05, ^**^represents p < 0.01, ^***^represents p < 0.001.

Furthermore, using the bootstrap method, we explore the mediating roles of self-oriented perfectionism and job crafting, as well as their chained mediating roles (Preacher and Hayes, 2004). As outlined in Table 5, the 95% confidence interval for the impact of self-oriented perfectionism is [0.016, 0.077] (excluding 0), indicating that self-oriented perfectionism significantly mediates the link between perceived overqualification and innovation performance, providing additional support for Hypothesis 2. Likewise, the 95% confidence interval for the effect of job crafting is [0.057, 0.189] (excluding 0), suggesting that job crafting significantly mediates the relationship between perceived overqualification and employee innovation performance, reinforcing Hypothesis 3. The 95% confidence interval for the joint effect of self-oriented perfectionism and job crafting is [0.001, 0.011] (excluding 0), indicating that self-oriented perfectionism and job crafting are chain mediators in linking perceived overqualification to employee innovation performance, confirming Hypothesis 4.

**Table 5:** Results of the mediation effect analysis.

**Action path**	**SE**	**Boot 95% CI**	
		**LLCI**	**ULCI**
Perceived overqualification → self-oriented perfectionism → innovation performance	0.016	0.016	0.077
Perceived overqualification → job crafting → innovation performance	0.034	0.057	0.189
Perceived overqualification → self-oriented perfectionism → job crafting → innovation performance	0.003	0.001	0.011

N = 363. CI represents confidence interval.

**Figure 2:**
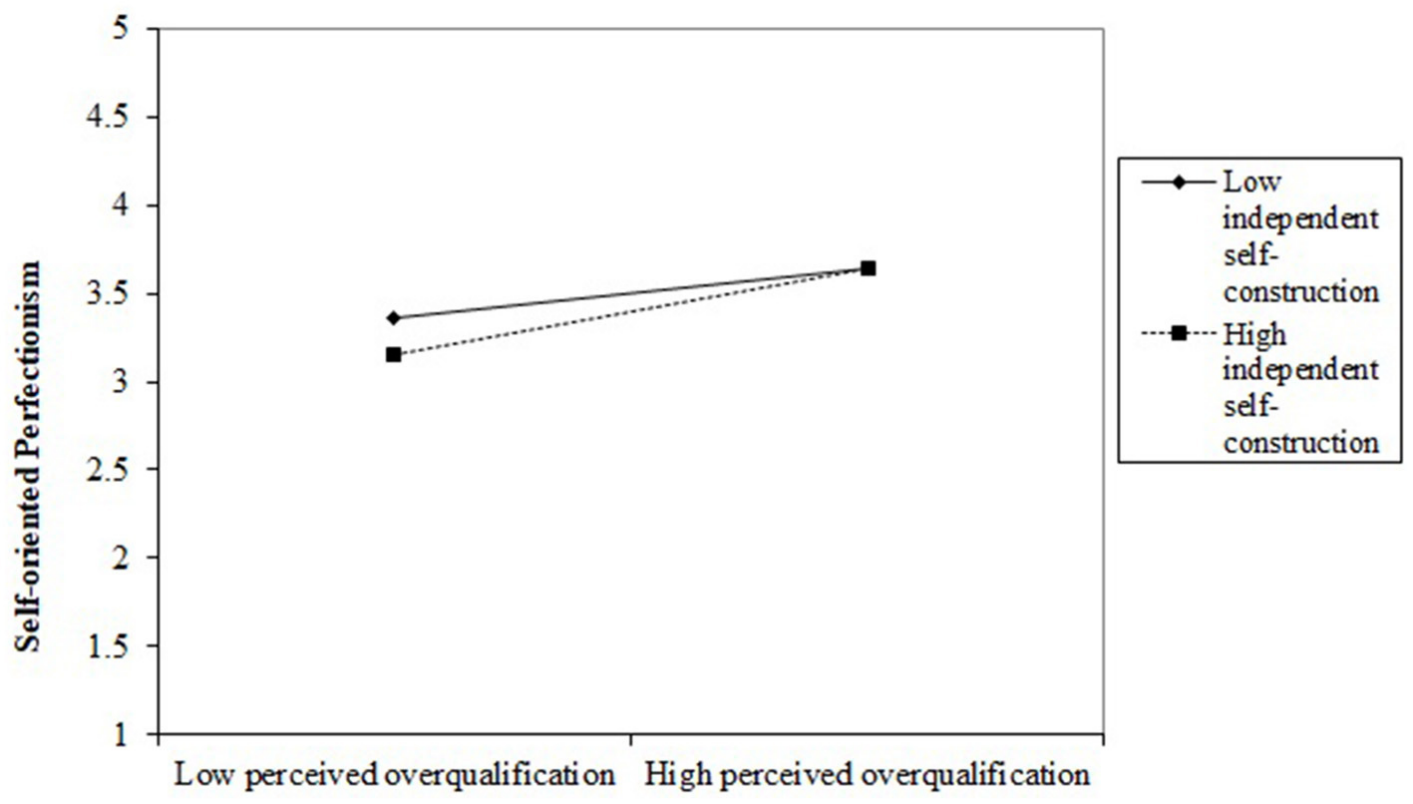
The interaction between perceived overqualification and independent self-construction on self-oriented perfectionism.

**Figure 3:**
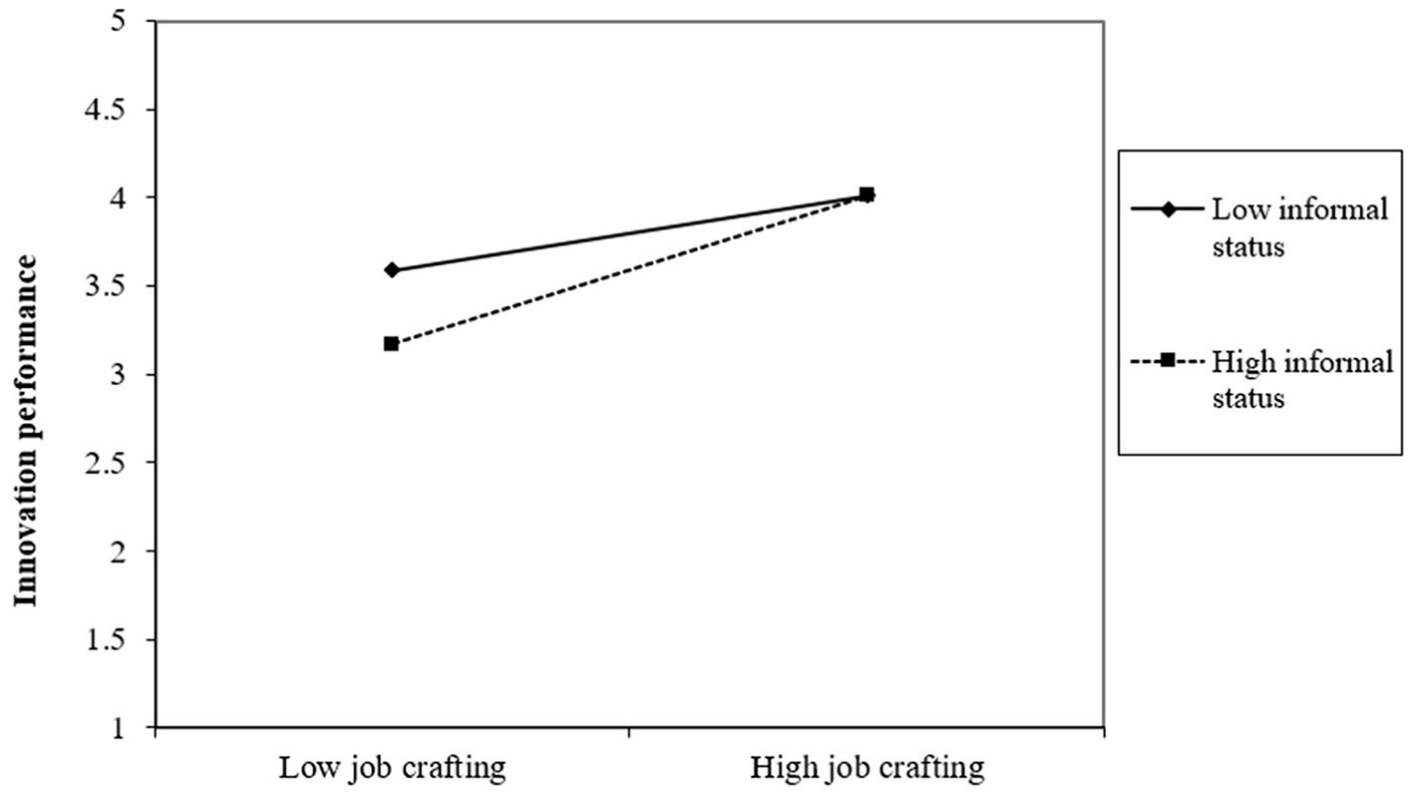
The interaction between job crafting and informal status on innovation performance.

To examine the moderating hypotheses (Hypotheses 5 and 7), we formulate two moderating models (Models 12 and 16), incorporating interaction terms between perceived overqualification and independent self-construction, as well as between job crafting and informal status, respectively. As depicted in Table 6, the interaction term between perceived overqualification and independent self-construction exhibits a positive association with self-oriented perfectionism (Model 12, r = 0.118, *p* < 0.05). This result implies that the positive link between perceived overqualification and self-oriented perfectionism is more pronounced among employees with higher levels of independent self-construction (refer to Figure 2), substantiating Hypothesis 5. Similarly, the interaction term between job crafting and informal status demonstrates a significant positive association with innovation performance (Model 16, r = 0.249, *p* < 0.001). The outcome indicates that the positive correlation between job crafting and innovation performance is heightened among individuals with elevated levels of informal status (refer to Figure 3), supporting Hypothesis 7.

**Table 6:** Results of hierarchical regression analysis.

	**Self-oriented perfectionism**				**Innovation performance**			
	**M 9**	**M 10**	**M 11**	**M 12**	**M 13**	**M 14**	**M 15**	**M 16**
Gender	−0.115	−0.121	−0.120	−0.111	0.010	−0.016	0.013	0.023
Age	−0.022	−0.021	−0.021^*^	0.020^*^	0.225^**^	0.212^**^	0.200^**^	0.213^**^
Education	−0.017	−0.014	−0.009	−0.007	−0.011	0.003	0.001	−0.001
Tenure	0.004^*^	0.003^*^	0.004	0.004	−0.401^***^	−0.366^***^	−0.344^***^	−0.376^***^
POQ		0.193^***^	0.183^***^	0.159^**^				
ISC			−0.100	−0.105				
POQ^*^ISC				0.118^*^				
JC						0.351^***^	0.327^***^	0.251^***^
IS							−0.167^***^	−0.209^***^
JC^*^IS								0.249^***^
R^2^	0.032	0.071	0.080	0.091	0.128	0.291	0.321	0.344
Δ R^2^	0.032	0.039	0.010	0.011	0.128	0.163	0.030	0.023
F-value	2.941	5.441	5.189	5.077	13.196	29.365	28.095	26.639

N = 363; POQ represents perceived overqualification; ISC represents independent self-construction; JC represents job crafting; IS represents informal status. ^*^Represents p < 0.05, ^**^represents p < 0.01, ^***^represents p < 0.001.

For a more in-depth examination of the moderating impacts of independent self-construction and informal status, we illustrate these effects through plotted figures, as depicted in Figures 2, 3. The slope of each line in the figures delineates the effects of perceived overqualification on self-oriented perfectionism across varying levels of independent self-construction and the influence of job crafting on innovation performance across different levels of informal status. The indicators Mean + SD (-SD) denote higher (lower) independent self-construction and informal status levels, respectively. Initially, when the level of independent self-construction is low, perceived overqualification positively affects self-oriented perfectionism (simple slope = 0.159, *p* < 0.001). Notably, this positive impact of perceived overqualification on self-oriented perfectionism becomes more pronounced at higher levels of independent self-construction (simple slope = 0.277, *p* < 0.001). Secondly, job crafting positively affects employee innovation performance when the level of informal status is low (simple slope = 0.251, *p* < 0.001). Significantly, this positive impact of job crafting on innovation performance is accentuated at higher levels of informal status (simple slope = 0.500, *p* < 0.001).

This study employs the bootstrap method to examine the moderating impact of independent self-construction and informal status on the chain-mediated relationship. As delineated in Table 7, at lower levels of independent self-construction and informal status, the indirect impacts of perceived overqualification on the innovation performance of employees through self-oriented perfectionism and job crafting are 0.009 and 0.067, with 95% confidence intervals of [-0.022, 0.048] (including 0) and [0.024, 0.120] (excluding 0), respectively. Conversely, at higher levels of independent self-construction and informal status, the indirect effects of perceived overqualification on employee innovation performance through self-oriented perfectionism and job crafting are 0.055 and 0.127, with 95% confidence intervals of [0.022, 0.095] (excluding 0) and [0.066, 0.228] (excluding 0), respectively. The between-group differences amount to 0.046 and 0.060, with 95% confidence intervals of [0.007, 0.058] (excluding 0) and [0.008, 0.138] (excluding 0), respectively, and the findings are statistically significant. These results underscore that the chain mediation of self-oriented perfectionism and job crafting varies across distinct levels of independent self-construction and informal status, indicating a moderation effect. Consequently, hypotheses 6 and 8 were substantiated.

**Table 7:** Results of the moderated mediating effect analysis.

**Moderator variables**	**Indirect effect**	**SE**	**Boot 95% CI**	
Low ISC (– SD)	0.009	0.017	−0.022	0.048
High ISC (+SD)	0.055	0.018	0.022	0.095
Difference (high ISC – low ISC)	0.046	0.013	0.007	0.058
Low IS (– SD)	0.067	0.025	0.024	0.120
High IS (+SD)	0.127	0.042	0.066	0.228
Difference (high IS – low IS)	0.060	0.033	0.008	0.138

N = 363; ISC represents independent self-construction; IS represents informal status. CI represents confidence interval.

## 5 Discussion

Leveraging the trait activation theory, the research develops a theoretical model to elucidate the relationship between perceived overqualification, self-oriented perfectionism, job crafting, and innovation performance. Furthermore, it examines the moderating influence of independent self-construction and informal status on this theoretical framework. The findings reveal that perceived overqualification significantly enhances employee innovation performance. Moreover, self-oriented perfectionism and job crafting are partial mediators in linking perceived overqualification to innovation performance. Additionally, self-oriented perfectionism positively influences job crafting, thereby acting as a chain mediating role. Lastly, independent self-construction moderates the relationship between perceived overqualification and self-oriented perfectionism, while informal status moderates the link between job crafting and employee innovation performance. Both factors jointly act as moderators between perceived overqualification and employee innovation performance.

### 5.1 Theoretical implications

Firstly, this study establishes that perceived overqualification can positively impact employee innovation performance, contributing a favorable outcome variable to the existing literature on overqualification. As a widespread phenomenon in contemporary management practice, previous studies have primarily examined the negative impacts of perceived overqualification through lenses such as person-job matching theory (Debus et al., 2020), equity theory (Cheng et al., 2020), and relative deprivation theory (Schreurs et al., 2021), it often overlooked the potential for employee initiative and creativity. In reality, employees with a strong sense of perceived overqualification possess knowledge and skills that exceed job requirements, which can be advantageous for the organization (Li et al., 2022). This study explores how subjective perceptions of overqualification can lead to enhanced innovation performance, providing a positive response to the academic research recommendations advocating for increased focus on the positive impacts of the perception of perceived overqualification (Russell et al., 2016).

Secondly, this research applies trait activation theory to elucidate the mechanism by which perceived overqualification influences outcomes, thereby enhancing the theoretical perspective within the overqualification domain. While existing studies have explored the positive effects of perceived overqualification through self-regulation theory and self-representation theory (Zhang et al., 2016; Erdogan et al., 2020), they have often neglected the activation effects of intrinsic motivational factors, such as employee competence, on employee traits. And we have found that an employee's intrinsic sense of satisfaction and achievement are crucial factors in realizing innovation performance (Elliot and Harackiewicz, 1994). Therefore, grounded in trait activation theory, this study verifies the chain-mediating roles of self-oriented perfectionism and job crafting in the relationship between perceived overqualification and innovation performance, responding to scholars' call for diverse perspectives on perceived overqualification in future research (Hu et al., 2015).

Thirdly, this study delves deeper into the boundary conditions shaping the impact of perceived overqualification on innovation performance through the moderating influence of independent self-construction and informal status. Previous research has shown that independent self-construction and informal status positively influence employees' innovation performance (Blackburn, 2005; Sun and Guo, 2021), primarily due to their strong psychological capital and relational resources. However, these studies often overlook the complexity of induced innovation behavior. By introducing independent self-construction and informal status as moderating variables, this study integrates independent self-construction with external environment perception and informal status with behavioral expression, providing a more comprehensive analysis of the boundary conditions influencing employees' innovation performance.

### 5.2 Practical implications

Initially, organizations ought to accurately discern the potential value embedded in overqualified employees and actively guide them to assess their perceived overqualification rationally. Employees harboring such perceptions possess extensive knowledge reserves and high technical proficiency, predisposing them to generate and implement innovative ideas. Enterprises must not only furnish ample opportunities and corresponding platforms but also proactively foster an optimistic work mindset among employees. This approach enables employees to view perceived overqualification as a potential asset, motivating them to channel their qualifications into creative endeavors, ultimately enhancing innovation performance.

Secondly, the research findings underscore that self-oriented perfectionism and job crafting behaviors significantly elevate employee innovation performance. Therefore, enterprises should prioritize managerial attention and support for employees with high perceived overqualification. By offering job opportunities, promotions, and future development prospects, companies can construct intrinsic motivation mechanisms that significantly stimulate self-oriented perfectionism, thereby fostering greater innovation. Moreover, organizations should empower employees by granting them greater autonomy in shaping work ideas and making decisions. Encouraging innovative behaviors through job crafting by creating a proactive organizational atmosphere and formulating effective incentive policies.

Thirdly, this study reveals that independent self-construction and informal status play pivotal roles in augmenting the impact of perceived overqualification on innovation performance. Managers should afford employees increased autonomy in work, empower them with decision-making authority and supportive conditions, provide substantial mental incentives, and help them shape an independent self-construction cognition. Additionally, attention should be directed toward formalizing the recognition of employees' informal status, tailoring measurement standards based on individual circumstances, and establishing a transparent management framework for cultivating informal status among employees. Collectively, these efforts enable employees to leverage their innovative behaviors more effectively.

### 5.3 Limitations and future directions

Firstly, it is noteworthy that all sample data are derived exclusively from Chinese enterprises, selectively excluding certain industries and regions characterized by distinct cultural and geographical attributes. While this approach may introduce certain limitations to the generalizability of the research findings, future studies can enhance their sample selection scope, ensuring a more comprehensive and reliable dataset. Secondly, this study delves into the indirect mechanism wherein perceived overqualification influences employee innovation performance, drawing insights from trait activation theory. Future exploration can delve deeper into whether perceived qualification among employees can extend their influence, empowering colleagues and organizations by sharing knowledge and information and enhancing overall effectiveness. Finally, this research incorporates independent self-construction and informal status as moderating variables, exclusively focusing on employees' internal factors. However, future investigations can benefit from examining the boundary conditions posed by external factors, such as leadership behavior and the organizational climate for error management, thereby contributing to a more nuanced understanding of the dynamics at play.

## 6 Conclusion

Drawing upon trait activation theory, this study investigates the mechanism and boundary conditions surrounding the impact of employees' perceived overqualification on their innovative performance in the workplace. This research holds both theoretical significance and practical insights. Companies should proactively encourage self-oriented perfectionism in employees who perceive themselves as overqualified to foster job crafting behaviors that enhance innovation performance. Such proactive measures can contribute to the long-term development of the company.

## Data availability statement

The raw data supporting the conclusions of this article will be made available by the authors, without undue reservation.

## Ethics statement

The studies involving humans were approved by School of Management, Shandong University of Technology. The studies were conducted in accordance with the local legislation and institutional requirements. The participants provided their written informed consent to participate in this study.

## Author contributions

HQ: Conceptualization, Data curation, Formal analysis, Methodology, Software, Writing – original draft, Writing – review & editing. BJ: Conceptualization, Data curation, Formal analysis, Funding acquisition, Methodology, Supervision, Writing – original draft, Writing – review & editing. SL: Data curation, Funding acquisition, Writing – review & editing. JZ: Data curation, Writing – review & editing.
